# Supplementary material for: Omics of an Enigmatic Marine Amoeba Uncovers Unprecedented Gene Trafficking from Giant Viruses and Provides Insights into Its Complex Life Cycle
Source: Microbiol Res (Pavia). Author manuscript; Available in PMC 2023 Sep 26. (PMC10521059; doi:10.3390/microbiolres14020047)
Supplement: Supplementary_material_Tekle [file NIHMS1925837-supplement-Supplementary_material_Tekle.zip › Supplementary_material_Tekle/electronic_supplementary_file_1_workflow.pdf]

Trichosphaerium Genome Assembly

1. Trim adapter using Trimmomatic v0.39

Bolger, A. M., Lohse, M., & Usadel, B. (2014). Trimmomatic: A flexible trimmer for Illumina Sequence Data. Bioinformatics, btu170.

https://github.com/usadellab/Trimmomatic.git

```
java -jar /usr/local/bin/Trimmomatic-0.39/trimmomatic-0.39.jar PE -threads 12 -phred33 /Volumes/Seagate
```

2. Assemble Long reads with Canu v2.2

Koren S, Walenz BP, Berlin K, Miller JR, Phillippy AM. Canu: scalable and accurate long-read assembly via adaptive k-mer weighting and repeat separation. Genome Research. (2017).

https://github.com/marbl/canu.git

```
/Users/teklelab/Documents/Softwares/canu/build/bin/canu -p Trichosphaerium_longreads -d Tricho-oxford g
```

3. Assess the genome assembly with BUSCO v5 eukaryote db

Use this tool: https://gvolante.riken.jp/analysis.html

4. Pilon v1.24 and bwa are used after assembly with Illumina short reads to polish (gap closing and error correcting) the assembly

Bruce J. Walker, Thomas Abeel, Terrance Shea, Margaret Priest, Amr Abouelliel, Sharadha Sakthikumar, Christina A. Cuomo, Qiandong Zeng, Jennifer Wortman, Sarah K. Young, Ashlee M. Earl (2014) Pilon: An Integrated Tool for Comprehensive Microbial Variant Detection and Genome Assembly Improvement. PLoS ONE 9(11): e112963. doi:10.1371/journal.pone.0112963

https://github.com/broadinstitute/pilon/wiki

Li H. and Durbin R. (2009) Fast and accurate short read alignment with Burrows-Wheeler Transform. Bioinformatics, 25:1754-60. [PMID: 19451168]

https://github.com/lh3/bwa.git

Li H., *Handsaker B.*, Wysoker A., Fennell T., Ruan J., Homer N., Marth G., Abecasis G., Durbin R. and 1000 Genome Project Data Processing Subgroup (2009) The Sequence alignment/map (SAM) format and SAMtools. Bioinformatics, 25, 2078-9. [PMID: 19505943]

https://github.com/samtools/samtools.git

```
###########################################################################################

Round 1: Original assembly from Canu

bwa index Trichosphaerium_longreads.contigs.fasta Trichosphaerium_longreads.contigs.fasta

bwa mem -t 10 -o Trichosphaerium_longreads.contigs_Illumina_mapping.out.sam Trichosphaerium_longreads.co

samtools view Trichosphaerium_longreads.contigs_Illumina_mapping.out.sam | samtools sort -o Trichosphaer

samtools index Trichosphaerium_longreads.contigs_Illumina_mapping.out.sorted.bam

pilon -Xmx100048m --genome Trichosphaerium_longreads.contigs.fasta --frags Trichosphaerium_longreads.con


###########################################################################################

Round 2: Pilon Polishes assembly from round 1

bwa index Tricho_Canu_longread_pilon1.fasta Tricho_Canu_longread_pilon1.fasta

bwa mem -t 10 -o Tricho_Canu_longread_pilon1_out.sam Tricho_Canu_longread_pilon1.fasta YT12_Tricho_R1.tr

samtools view Tricho_Canu_longread_pilon1_out.sam | samtools sort -o Tricho_Canu_longread_pilon1_out.sor


samtools index Tricho_Canu_longread_pilon1_out.sorted.bam

pilon -Xmx100048m --genome Tricho_Canu_longread_pilon1.fasta --frags Tricho_Canu_longread_pilon1_out.sor

###########################################################################################


Round 3: Pilon Polishes assembly from round 2

bwa index Tricho_Canu_longread_pilon2.fasta Tricho_Canu_longread_pilon2.fasta

bwa mem -t 10 - Tricho_Canu_longread_pilon2.sam Tricho_Canu_longread_pilon2.fasta YT12_Tricho_R1_001_tri

samtools view  Tricho_Canu_longread_pilon2.sam | samtools sort -o Tricho_Canu_longread_pilon2.sorted.bam

samtools index Tricho_Canu_longread_pilon2.sorted.bam

pilon -Xmx100048m --genome Tricho_Canu_longread_pilon2.fasta  --frags Tricho_Canu_longread_pilon2.sorted
```

5. Contaminant removal with Blobtoolkit v3

Kumar et al. 2013. Blobology: exploring raw genome data for contaminants, symbionts and parasites using taxon-annotated GC-coverage plots. Frontiers in Genetics, 4:237

Laetsch & Blaxter 2017. BlobTools: Interrogation of genome assemblies [version 1; referees: awaiting peer review]. F1000Research, 6:1287 (doi: 10.12688/f1000research.12232.1)

https://blobtoolkit.genomehubs.org/install/

```
diamond blastx \
        --query Tricho_Canu_longread_pilon3.sorted_v1.fa \
        --db /Volumes/Seagate_Backup/nr.dmnd \
        --outfmt 6 qseqid staxids bitscore qseqid sseqid pident length mismatch gapopen qstart qend ssta
        --sensitive \
        --max-target-seqs 1 \
        --evalue 1e-3 \
        --threads 16 \
        > Tricho_diamond.out.txt
```

1. Use diamond blastx to search the assembly contigs against nt database

2. Use minimap2 to map all Illumina reads the reference assembly (reference should be provided first)

- Short reads:

```
minimap2 -a -x sr Tricho_Canu_longread_pilon3.sorted_v1.fa YT12_Tricho_R1.trimmomatic.50x.50x.fastq.gz \
```

```
samtools view Tricho_longreads_mapped.sam | samtools sort -o Tricho_shortreads_mapped_combined_sorted.ba
```

3. Convert sam to bam and merge and sort the files

4. Radundans to reduce the heterozygous regions in the genome

Leszek P. Pryszcz and Toni Gabaldón (2016) Redundans: an assembly pipeline for highly heterozygous genomes. NAR. doi: 10.1093/nar/gkw294

https://github.com/lpryszcz/redundans.git

```
python2 /Users/teklelab/Documents/Softwares/redundans/redundans.py -f Tricho_Canu_longread_pilon3.sorte
```

5. Running blobtools

- Create a new BlobDir from a FASTA file:

```
blobtools add --fasta Tricho_Canu_longread_pilon3.sorted_v1.fa Tricho_blobtool
```

- Add BLAST hits

```
blobtools add \
    --hits Tricho_diamond.out.txt \
    --taxrule bestsumorder \
    --taxdump  /Users/teklelab/Documents/Softwares/new_taxdump \
     Tricho_blobtool
```

- Add mapping coverage

```
blobtools  add \
    --cov Tricho_shortreads_mapped_combined_sorted.bam \
    Tricho_blobtool
```

- Add BUSCO scores (remember to remove all missing genes in the table)

```
blobtools add \
    --busco Tricho_blobtool/Tricho_busco/Tricho_busco.txt \
    Tricho_blobtool
```

- Open dataset in BlobToolKit Viewer

```
blobtools host `pwd`
```

- Select the contigs that met the following criteria: (1) taxonomic assignment to bacteria, archaea, or viruses, (2) low or high GC percentage indicative of organellar scaffolds or contaminated contigs, and (3) don't have BUSCO gene assigned.

6. Annotation with BRAKER2

- Clean up genome headers

```
####Replace definition lines. Utility script in: https://github.com/josephryan/JFR-PerlModules

#First determine the pad value by running:

grep -c '^>' blah.gte200.fa | perl -ne '$num = scalar(split/|/); print "$num\n";'

#Then replace the deflines using the pad value

replace_deflines.pl --fasta=blah.gte200.fa --prefix=blah --pad=5 > Tricho_Canu_longread_pilon3.sorted_v1

# Add number of bases in header
cat Tricho_Canu_longread_pilon3.sorted_v1.fa | seqkit fx2tab --length | awk -F "\t" '{print $1"_"$4"\t"$
```

- Align RNA-seq of Trichosphaerium into the decontaminated assembly with STAR

```
##create indexed genome
/ocean/projects/tra180030p/ytekle/Software/STAR/bin/Linux_x86_64_static/STAR \
--runMode genomeGenerate \
--genomeDir ./genome-index \
--genomeFastaFiles Tricho_Canu_longread_pilon3.sorted_v1.fa

##align RNA-seq reads to the assembly
/jet/home/ytekle/anaconda3/bin/STAR \
--genomeDir ./genome-index \
--readFilesIn YT42_R1_fwd_combined.fastq,YT42_R2_rev_combined.fastq \
--outSAMtype BAM SortedByCoordinate
```

- Create ProHint file using A. casstellanii proteome file

```
/ocean/projects/tra180030p/ytekle/Software/ProtHint/bin/prothint.py \
/ocean/projects/tra180030p/ytekle/htran/Tricho_SPADES_assembly/09-Tricho-longreads-pilon/Tricho_Canu_lon
/ocean/projects/tra180030p/ytekle/htran/N5_spades_nanopore_assembly/N5_genome_annotation/00-DATA/GCF_000
--workdir /ocean/projects/tra180030p/ytekle/htran/Tricho_SPADES_assembly/09-Tricho-longreads-pilon/
```

- Run Braker using the bam alignment file and ProHint outout from above

```
PATH=/ocean/projects/tra180030p/ytekle/Software/BRAKER/scripts/:$PATH
export PATH

braker.pl --genome=/ocean/projects/tra180030p/ytekle/htran/Tricho_SPADES_assembly/09-Tricho-longreads-pi
--bam=/ocean/projects/tra180030p/ytekle/htran/Tricho_SPADES_assembly/09-Tricho-longreads-pilon/Tricho_RN
--hints=/ocean/projects/tra180030p/ytekle/htran/Tricho_SPADES_assembly/09-Tricho-longreads-pilon/prothin
--etpmode \
--BAMTOOLS_PATH=/ocean/projects/tra180030p/ytekle/Software/bamtools/bin \
--cores=8
```
