# Supplementary figures and images for: Omics of an Enigmatic Marine Amoeba Uncovers Unprecedented Gene Trafficking from Giant Viruses and Provides Insights into Its Complex Life Cycle
Source: Microbiol Res (Pavia). Author manuscript; Available in PMC 2023 Sep 26. (PMC10521059; doi:10.3390/microbiolres14020047)

COGs Distribution among LGT gene candidates

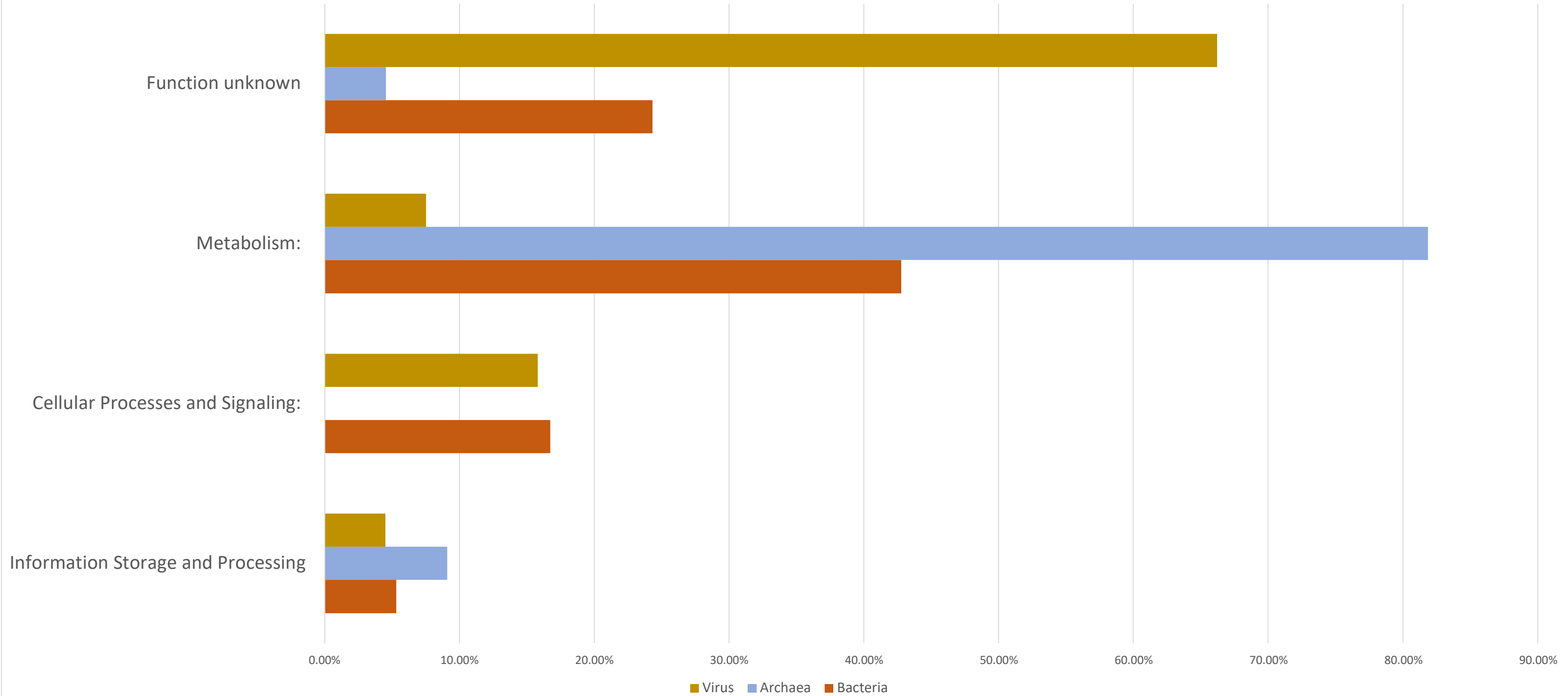

Supplement: Supplementary_material_Tekle [file NIHMS1925837-supplement-Supplementary_material_Tekle.zip › Supplementary_material_Tekle/Fig_S2.pdf]

A

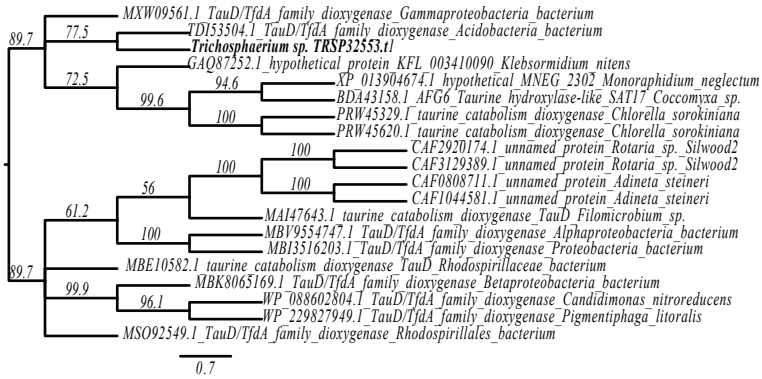

B

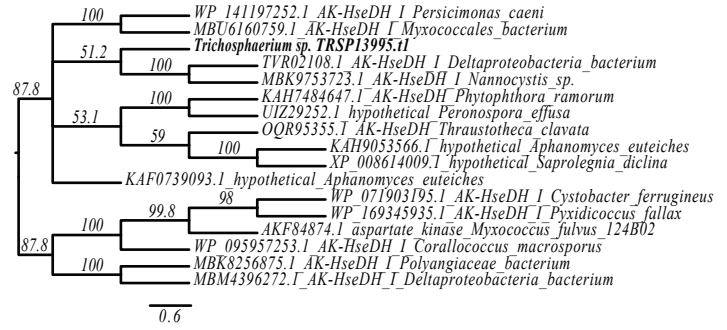

C

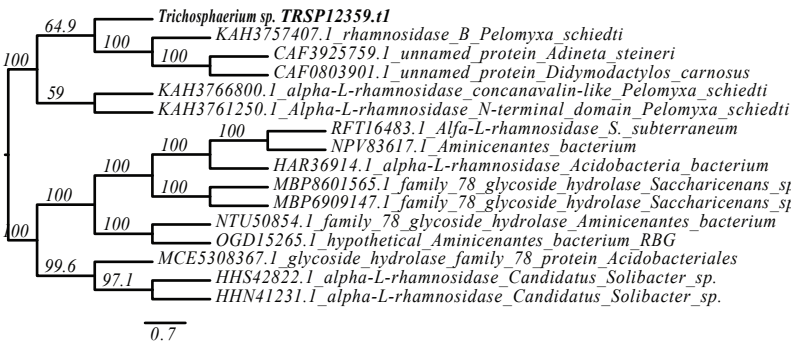

D

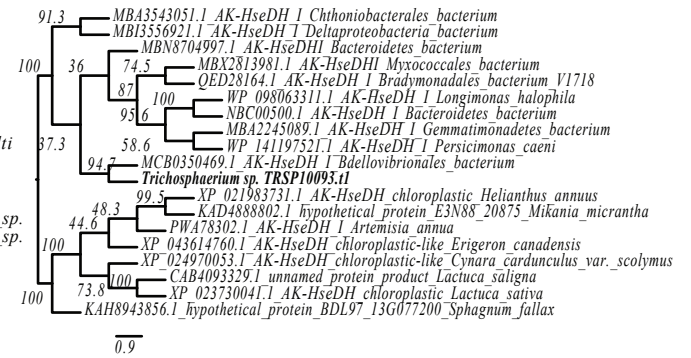

E

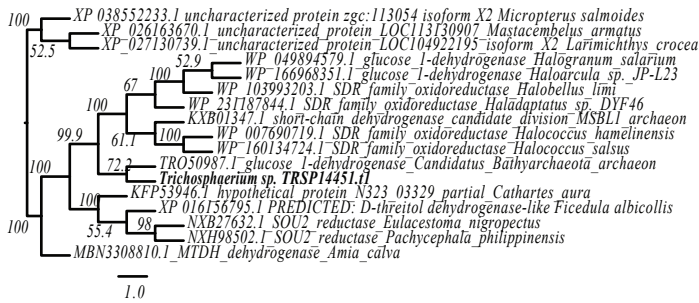

F

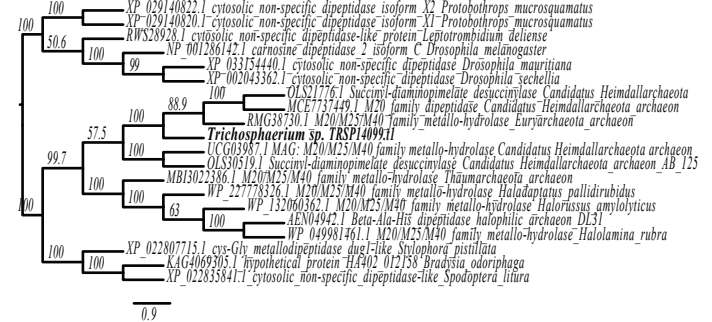

G

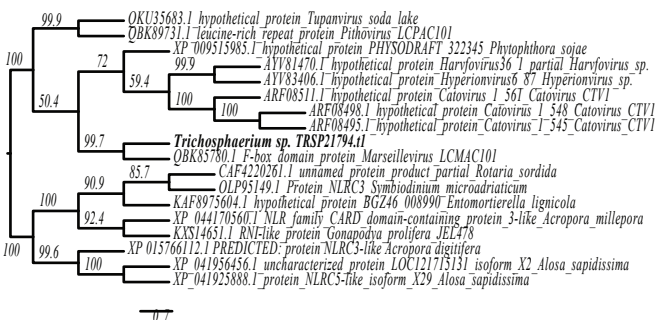

H

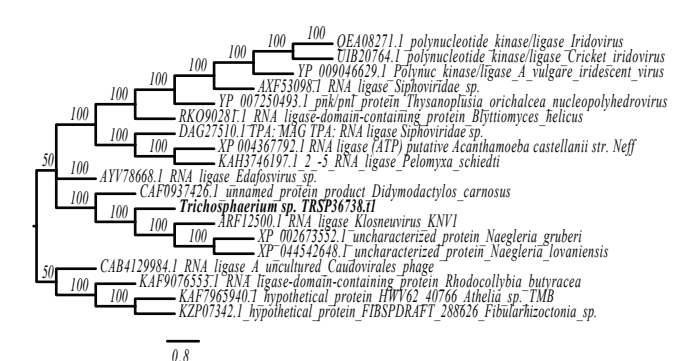

Supplement: Supplementary_material_Tekle [file NIHMS1925837-supplement-Supplementary_material_Tekle.zip › Supplementary_material_Tekle/Fig_S3_LGT_A_h.pdf]

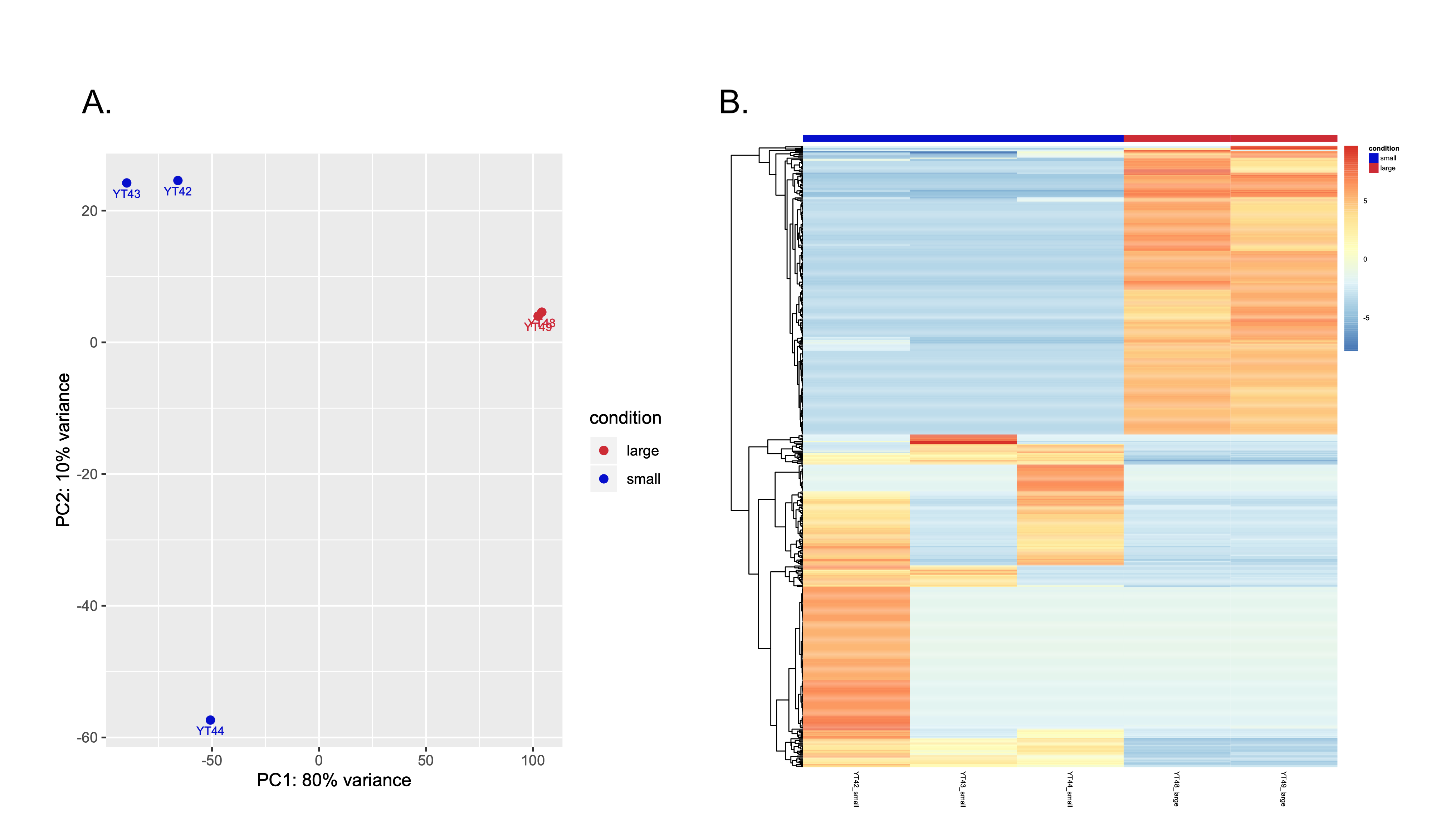

Supplement: Supplementary_material_Tekle [file NIHMS1925837-supplement-Supplementary_material_Tekle.zip › Supplementary_material_Tekle/Fig_S5_DGE_2X.png]

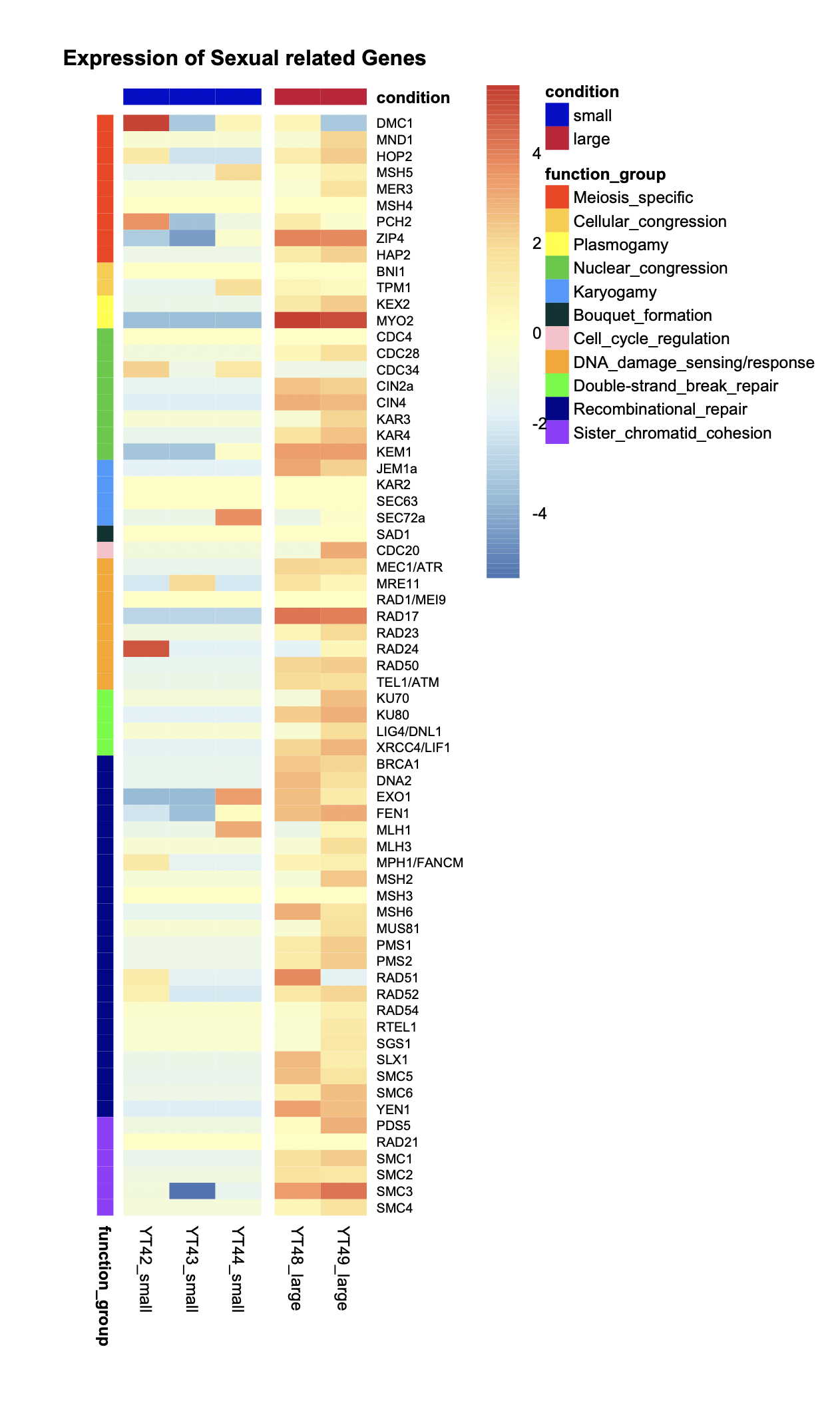

Supplement: Supplementary_material_Tekle [file NIHMS1925837-supplement-Supplementary_material_Tekle.zip › Supplementary_material_Tekle/Fig_S7_dge_sex.png]

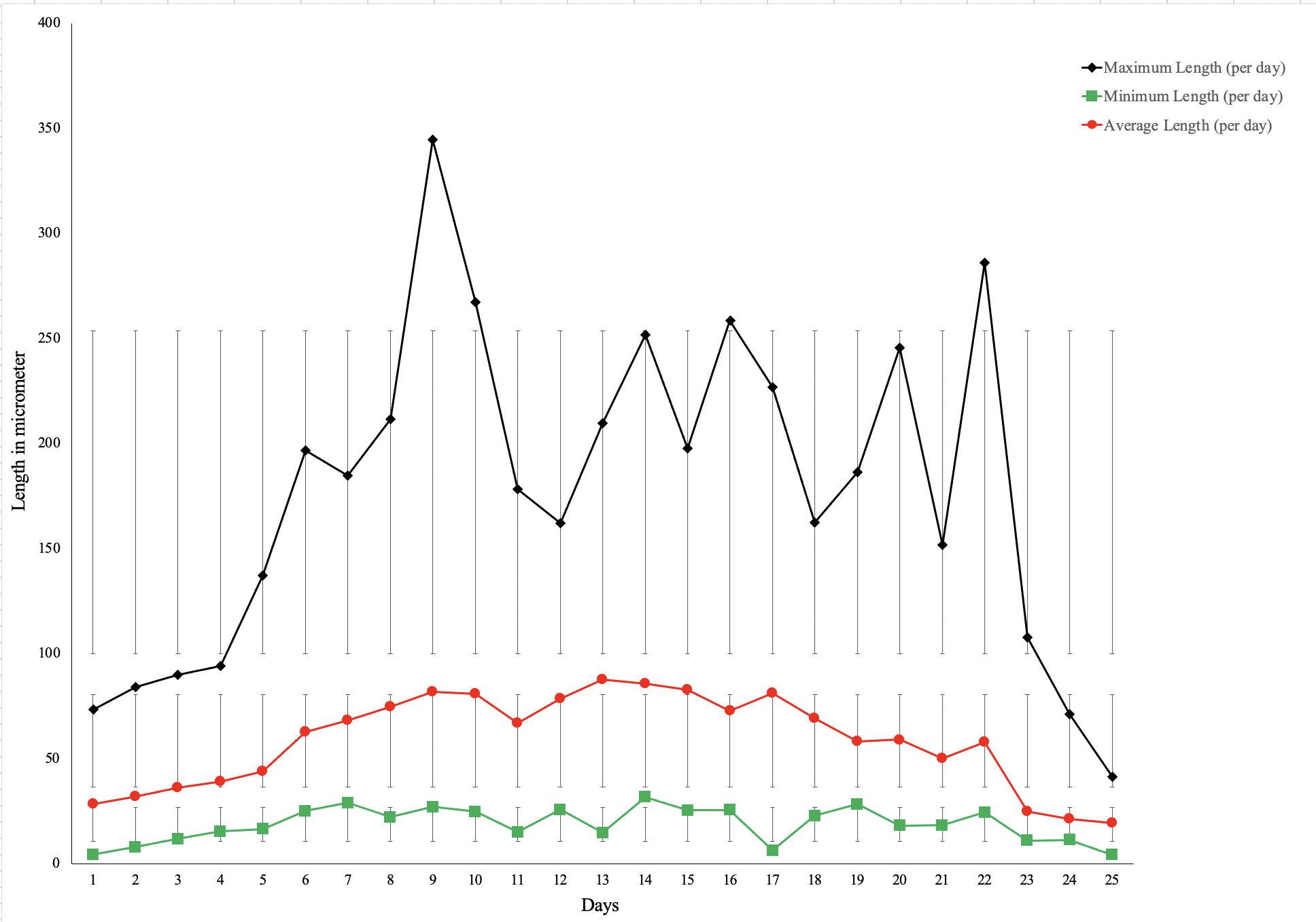

Supplement: Supplementary_material_Tekle [file NIHMS1925837-supplement-Supplementary_material_Tekle.zip › Supplementary_material_Tekle/Fig_S1_life_cycle_fig_new.png]

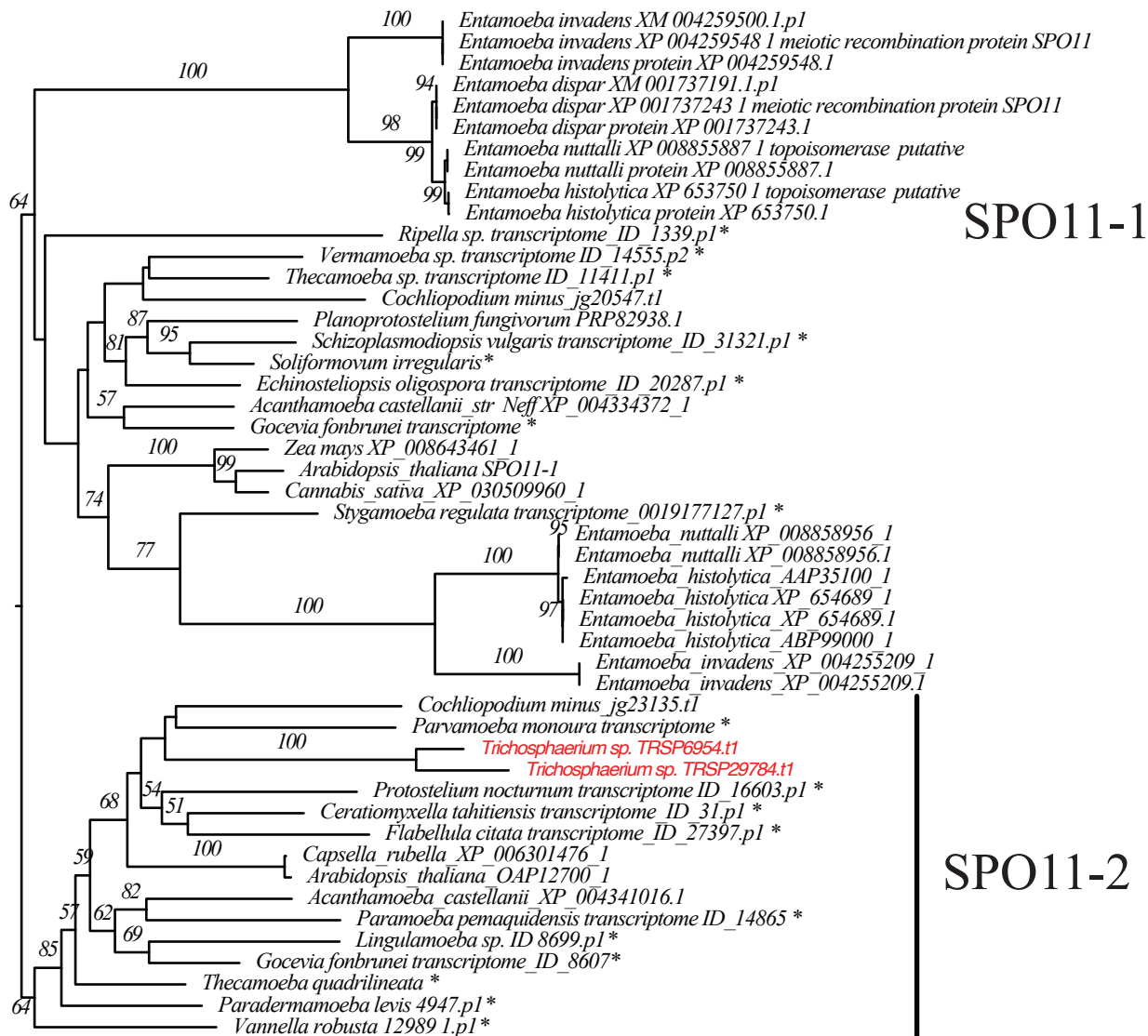

Supplement: Supplementary_material_Tekle [file NIHMS1925837-supplement-Supplementary_material_Tekle.zip › Supplementary_material_Tekle/Fig_S8_spo11_1_2_combo_NEW.pdf]

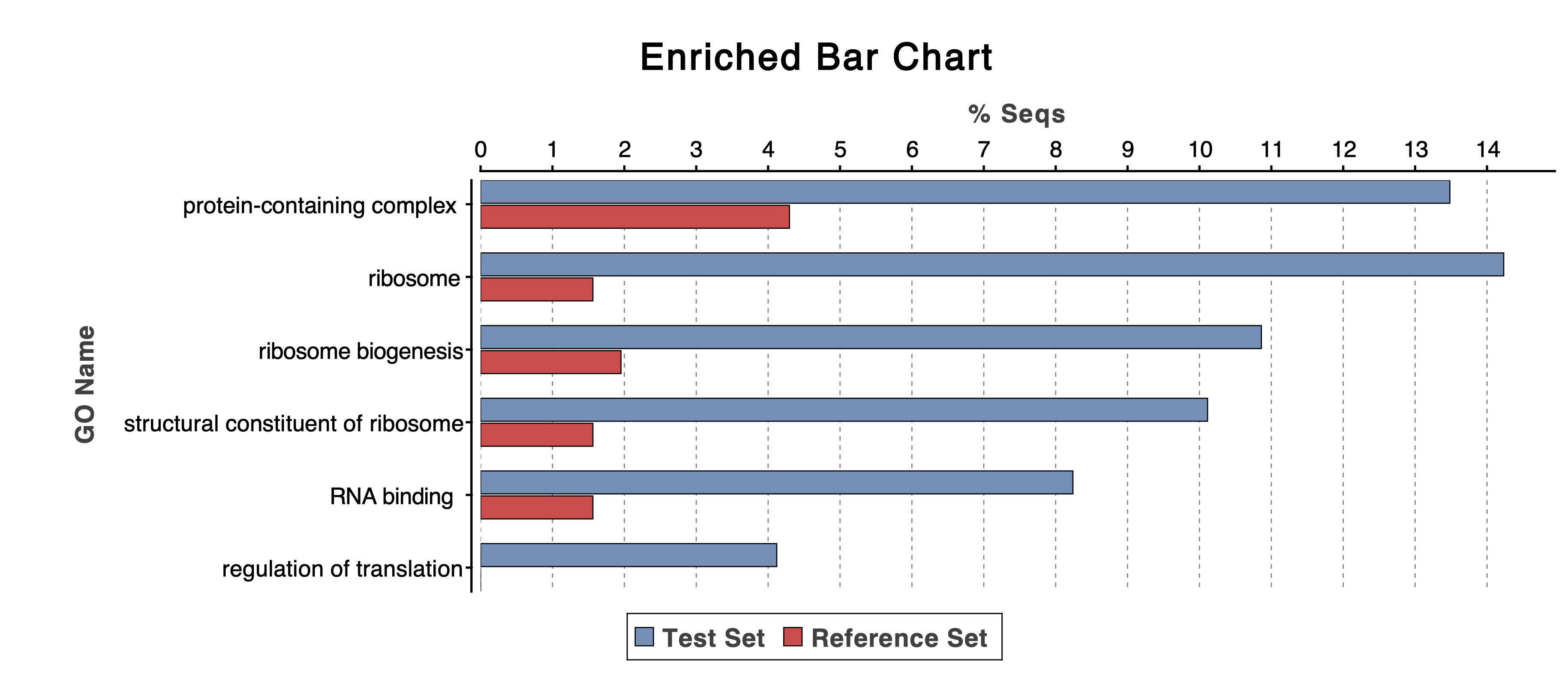

Supplement: Supplementary_material_Tekle [file NIHMS1925837-supplement-Supplementary_material_Tekle.zip › Supplementary_material_Tekle/Fig_S6_DGE_ls.png]

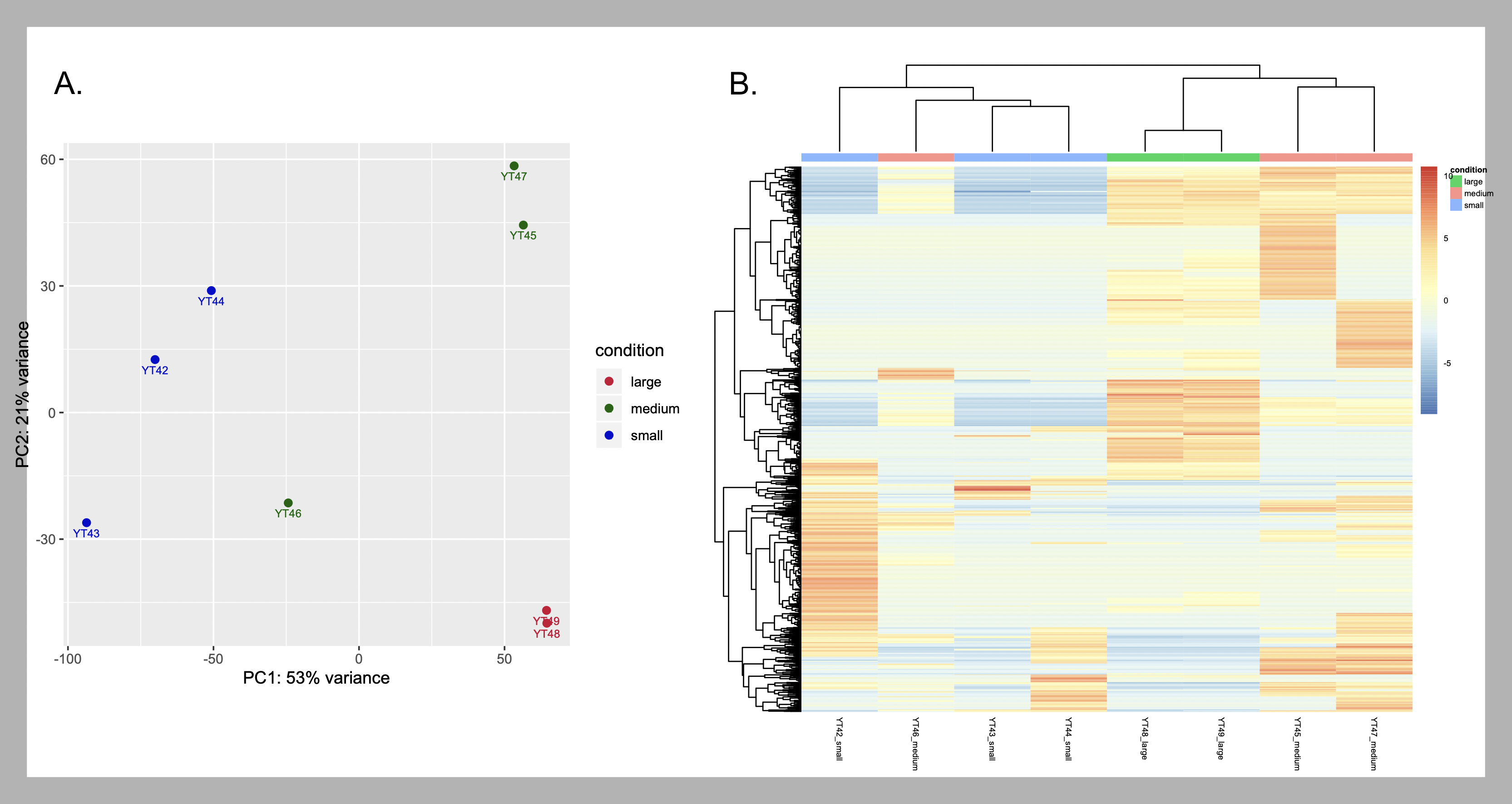

Supplement: Supplementary_material_Tekle [file NIHMS1925837-supplement-Supplementary_material_Tekle.zip › Supplementary_material_Tekle/Fig_S4_DGE_3X.png]
